# Amount and pattern of physical activity and sedentary behavior are associated with kidney function and kidney damage: The Maastricht Study

**DOI:** 10.1371/journal.pone.0195306

**Published:** 2018-04-04

**Authors:** Remy J. H. Martens, Julianne D. van der Berg, Coen D. A. Stehouwer, Ronald M. A. Henry, Hans Bosma, Pieter C. Dagnelie, Martien C. J. M. van Dongen, Simone J. P. M. Eussen, Miranda T. Schram, Simone J. S. Sep, Carla J. H. van der Kallen, Nicolaas C. Schaper, Hans H. C. M. Savelberg, Frank M. van der Sande, Abraham A. Kroon, Jeroen P. Kooman, Annemarie Koster

**Affiliations:** 1 Department of Internal Medicine, Maastricht University Medical Center+, Maastricht, the Netherlands; 2 NUTRIM School for Nutrition and Translational Research in Metabolism, Maastricht University, Maastricht, the Netherlands; 3 Department of Social Medicine, Maastricht University, Maastricht, the Netherlands; 4 CAPHRI Care and Public Health Research Institute, Maastricht University, Maastricht, the Netherlands; 5 CARIM School for Cardiovascular Diseases, Maastricht University, Maastricht, the Netherlands; 6 Heart and Vascular Centre, Maastricht University Medical Center+, Maastricht, the Netherlands; 7 Department of Epidemiology, Maastricht University, Maastricht, the Netherlands; 8 Department of Human Movement Sciences, Maastricht University, Maastricht, the Netherlands; Hospital Universitario de la Princesa, SPAIN

## Abstract

**Background:**

Chronic kidney disease, which is defined as having a reduced kidney function (estimated glomerular filtration rate (eGFR)) and/or signs of kidney damage (albuminuria), is highly prevalent in Western society and is associated with adverse health outcomes, such as cardiovascular disease. This warrants a search for risk factors of lower eGFR and higher albuminuria. Physical activity and sedentary behavior may be such risk factors.

**Objective:**

To examine associations of physical activity (total, high, low), sedentary time and sedentary behavior patterns (breaks, prolonged bouts, average bout duration) with eGFR and albuminuria.

**Methods:**

We examined these associations in 2,258 participants of the Maastricht Study (average age 60.1±8.1 years; 51.3% men), who wore an accelerometer 24h/day on 7 consecutive days. Associations with continuous eGFR and categories of urinary albumin excretion (UAE; <15 [reference category], 15-<30, ≥30 mg/24h) were evaluated with linear regression analyses and multinomial logistic regression analyses, respectively.

**Results:**

After adjustment for potential confounders, each extra hour of total physical activity was associated with a more favorable kidney function (beta_eGFR_ = 2.30 (95%CI = 1.46; 3.14)), whereas each extra hour of sedentary behavior was associated with a more adverse kidney function (beta_eGFR_ = -0.71 (-1.08; -0.35)). Also, compared to individuals with the lowest levels of total physical activity, individuals with the highest levels had less kidney damage (OR_15-<30mg/24h_ = 0.63 (0.41; 0.96), OR_≥30mg/24h_ = 0.84 (0.53; 1.35). An extra hour of sedentary behavior was associated with more kidney damage (OR_15-<30 mg/24h_ = 1.11 (1.01; 1.22), OR_≥30 mg/24h_ = 1.10 (0.99; 1.22)). Further, a highly sedentary pattern was associated with a more adverse kidney function, but no association was seen with kidney damage.

**Conclusions:**

Physical activity and sedentary behavior were associated with kidney function and kidney damage. Additionally, sedentary behavior patterns were associated with kidney function. Causal studies are required to examine whether this indeed implicates that prevention strategies should focus not only on increasing physical activity, but on reducing sedentary behavior as well.

## Introduction

Reduced kidney function (estimated glomerular filtration rate (eGFR) <60 ml/min/1.73m^2^) and kidney damage (albuminuria ≥30 mg/24h), which together define chronic kidney disease (CKD), have become highly prevalent in modern Western society [1]. Both have been associated with end-stage renal disease [2], acute kidney injury [3], and cardiovascular disease (CVD) risk [2]. Therefore, it is important to identify factors that contribute to the development of reduced eGFR and albuminuria, which are amenable for intervention.

A possibly important factor is physical activity, which includes not only moderate to vigorous physical activity (MVPA) like brisk walking or running, but also light intensity physical activity such as casual walking and household work [4]. Further, adults spend most time of the day in sedentary behaviors [5–8], such as watching TV, using the computer or driving. Sedentary behavior has been associated with metabolic and CVD risk factors, independent of physical activity [9]. Therefore, sedentary behavior should be examined next to the different levels of physical activity. Several studies have reported on associations of physical activity [10–15] and sedentary behavior [12,16] with eGFR [10,12,14,15], albuminuria [11–13,16], or both combined as CKD [12]. However, these studies have used self-reported measures of physical activity and sedentary behavior, which may be biased and the reported results have been inconsistent.

Since several years the complete daily activity spectrum, from sedentary behavior to MVPA, can be objectively measured with an accelerometer. To date only a few studies have described associations between accelerometry data and kidney function [17,18]. These studies have not reported on the pattern of sedentary time, *i*.*e*., the manner in which sedentary time was accumulated. Since not all sedentary time is bad (certain amounts are needed for rest and recovery) and particularly prolonged uninterrupted sedentary time may be harmful, sedentary patterns should be examined. These patterns can be expressed by sedentary breaks, which refers to the frequency with which sedentary time was interrupted (transitions from sitting to standing) and sedentary bouts, which refers to uninterrupted periods of sedentary time of a certain duration (*e*.*g*., 30 min). Previous studies have demonstrated that sedentary patterns were associated with detrimental health outcomes, including a larger waist circumference and a higher body mass index, higher levels of triglycerides and glucose, and the metabolic syndrome [19,20].

Therefore, we measured total amount and patterns of physical activity and sedentary behavior with an accelerometer in a large sample of adults aged 40–75 years who participated in The Maastricht Study. The aim of this study was to examine associations of physical activity (total, high, low), sedentary time and sedentary behavior patterns (breaks, prolonged bouts, average bout duration) with eGFR and albuminuria.

## Materials and methods

### Study population and design

We used data from The Maastricht Study, an observational prospective population-based cohort study. The rationale and methodology have been described previously [21]. In brief, the study focuses on the etiology, pathophysiology, complications and comorbidities of type 2 diabetes mellitus (T2DM) and is characterized by an extensive phenotyping approach. Eligible participants were all individuals aged between 40 and 75 years and living in the southern part of the Netherlands. Participants were recruited through mass media campaigns and from the municipal registries and the regional Diabetes Patient Registry via mailings. Recruitment was stratified according to known T2DM status, with an oversampling of individuals with T2DM, for reasons of efficiency. The present report includes cross-sectional data from the first 3,451 participants, who completed the baseline survey between November 2010 and September 2013. The examinations of each participant were performed within a time window of three months.

For the present study, we used complete cases, so participants were excluded when: having another diabetes type than T2DM (n = 41); they did not receive an accelerometer due to logistics (n = 668), their accelerometer measurement failed (n = 135), eGFR was missing (n = 20), 24h urine collections were either collected erroneously (<20h or >28h) or were not handed in at all (n = 23), or having other missing data (n = 306). A total of 2,258 participants were included in the present study.

The study has been approved by the institutional medical ethical committee (NL31329.068.10) and the Minister of Health, Welfare and Sports of the Netherlands (Permit 131088-105234-PG), and was conducted in accordance with the Declaration of Helsinki. All participants gave written informed consent.

### Materials

#### Accelerometry data

Daily activity levels were measured using the activPAL3^™^ physical activity monitor (PAL Technologies, Glasgow, UK), as described elsewhere [20]. The device was attached directly to the skin in front of the right thigh, after the device had been waterproofed. Participants were asked to wear the accelerometer for 8 consecutive days, without removing it at any time. Data from the first day were excluded from the analysis because participants performed physical function tests at the research center after the device was attached. Participants were included if they provided at least 1 valid day (≥10h of waking data).

The total amount of physical activity was based on the stepping posture and calculated as the mean time spent in stepping during waking time per day. The method used to determine waking time has been described elsewhere [22]. Physical activity (stepping time) was further classified into higher intensity physical activity (HPA; minutes with a step frequency >110 steps/min during waking time) and lower intensity physical activity (LPA; minutes with a step frequency ≤110 steps/min during waking time; standing time was not included). The total amount of sedentary time was based on the sedentary posture (sitting/lying), and calculated as the mean time spent in a sedentary position during waking time per day. Three constructs of sedentary behavior patterns were identified: number of sedentary breaks, number prolonged sedentary bouts, and average sedentary bout duration. The number of sedentary breaks during waking time was determined as each transition from a sitting/lying position to standing or stepping with a duration of at least 1 min, and the mean number of breaks/day was calculated. Sedentary time accumulated in a consecutive period ≥30 min was defined as a prolonged sedentary bout, and the mean number of prolonged sedentary bouts/day was calculated. Average bout duration was calculated by dividing total sedentary time by total number of sedentary bouts of any duration.

#### Kidney function

GFR was estimated with the Chronic Kidney Disease Epidemiology Collaboration (CKD-EPI) equation based on the combination of serum creatinine and serum cystatin C (eGFR_crcys_) [23]. Serum creatinine and serum cystatin C were assessed as described elsewhere [24]. To assess urinary albumin excretion (UAE), participants were requested to collect two 24h urine collections, as described elsewhere [24]. Only urine collections with a collection time between 20h and 28h were considered valid. If needed, UAE was extrapolated to a 24h excretion. These analyses were preferably based on the average of two (available in 92.6% of the participants) 24h urine collections.

#### Potential confounders or mediators

Potential confounders or mediators were extracted from questionnaires and included sex, age, smoking behavior, alcohol consumption, daily energy intake, mobility limitation (having any difficulties with walking in the previous week), noncardiovascular comorbidity (the presence of (a history of) non-skin cancer, inflammatory respiratory disease or Parkinson’s disease), and history of CVD. Level of education and use of antihypertensive and lipid-modifying medication were assessed by interview.

Other potential confounders or mediators were obtained from physical examination and laboratory assessment as described elsewhere [21], and included waist circumference, total cholesterol, high-density lipoprotein (HDL)-cholesterol, triglycerides, office blood pressure, 24h average ambulatory blood pressure, and glucose metabolism status.

### Statistical analyses

Characteristics of the total study population and according to sex-specific quartiles of sedentary time (to obtain equal distributions of men and women) were summarized as means with standard deviations (SD) or as numbers and percentages. Non-normally distributed variables were described using the median and the interquartile range.

General linear models were used to obtain adjusted levels of physical activity and sedentary behavior variables according to categories of eGFR_crcys_ and albuminuria. eGFR_crcys_ was categorized into: ≥90 ml/min/1.73m^2^, 60-<90 ml/min/1.73m^2^, and <60 ml/min/1.73m^2^ [24]. Albuminuria was categorized into: <15 mg/24h, 15-<30 mg/24h, and ≥30 mg/24h [24].

Associations of total physical activity (h/day), HPA (10 min/day), LPA (h/day), sedentary time (h/day), sedentary breaks (10/day), prolonged sedentary bouts (#/day), and average sedentary bout duration (min) with eGFR_crcys_ (ml/min/1.73m^2^) as dependent variable were evaluated with multivariable linear regression analyses. The associations of the physical activity and sedentary behavior variables with albuminuria were evaluated with categories of albuminuria using multinomial logistic regression analyses, because UAE was highly positively skewed and could not be transformed adequately using common transformations [25]. Total physical activity and LPA were categorized into quartiles as the association with categorical albuminuria was non-linear.

The associations in models 1 were adjusted for the following variables which may confound associations of physical activity and sedentary behavior with kidney function and kidney damage: age, sex, glucose metabolism status, educational level (as an indicator of socioeconomic status), smoking status, alcohol consumption, energy intake, noncardiovascular comorbid disease, mobility limitation, and waking time (to exclude the possibility that estimated effects were biased due to differences in waking hours). In models 2 the associations with the sedentary behavior variables were additionally adjusted for HPA, to examine whether the effects were independent of the amount of HPA since HPA has been identified as an important health factor [26]. In models 3 the associations with HPA and the sedentary pattern variables were additionally adjusted for sedentary time, to examine whether the effects were independent of the amount of sedentary time. In models 4 all associations were additionally adjusted for variables which may confound but also mediate associations of physical activity and sedentary behavior with kidney function and kidney damage. These variables included office systolic blood pressure, use of antihypertensive medication, waist circumference, total-to-HDL cholesterol ratio, triglycerides, use of lipid-modifying medication, and prevalent CVD. Glucose metabolism status may also be a mediator in the associations examined. However, to control for the oversampling of individuals with T2DM in The Maastricht Study, glucose metabolism status was added in models 1. In an additional analyses, we repeated models 1 without adjustment for glucose metabolism status.

Variance inflation factors were <3.5 for all non-multiplicative variables, indicating absence of multicollinearity.

All analyses were performed with IBM SPSS Statistics Version 22.0 (IBM Corp., Armonk, NY, USA).

## Results

### Population characteristics

**Fig 1:**
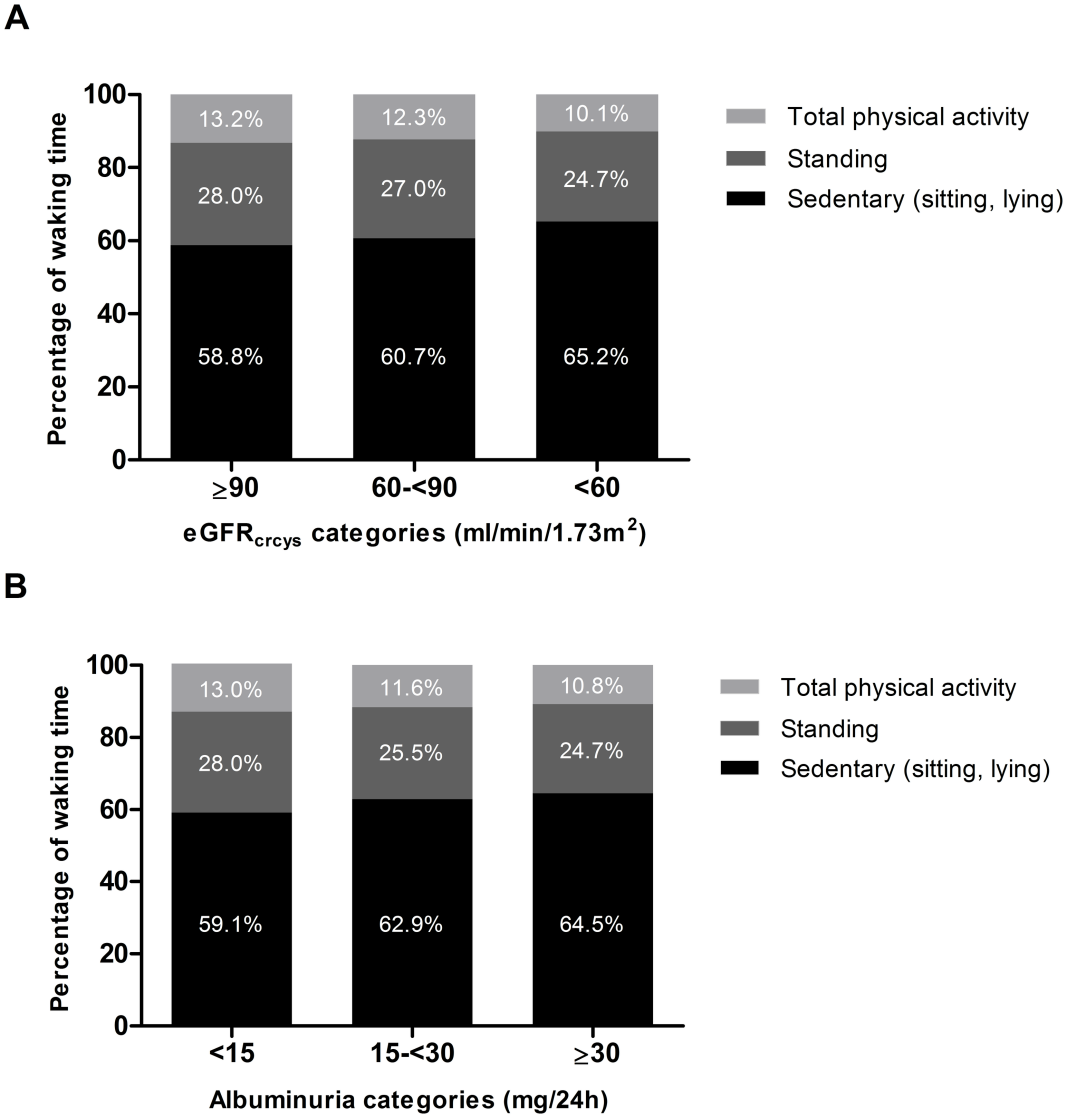
Percentage of waking time spent sedentary, standing, and physically active according to categories of eGFR_crcys_ (panel A) and albuminuria (panel B). Distribution of participants in the eGFR_crcys_ categories: ≥90 ml/min/1.73m^2^ n = 1073, 60-<90 ml/min/1.73m^2^ n = 1077, <60 ml/min/1.73m^2^ n = 97. Distribution of participants in the albuminuria categories: <15 mg/24h n = 1804, 15-<30 mg/24h n = 246, ≥30 mg/24h n = 197.

Of the total study population, 51.3% were men and the average age was 60.1±8.1 years (Table 1). eGFR_crcys_ was on average 87.9±14.6 ml/min/1.73m^2^ in the total study population, and 4.3% had an eGFR_crcys_ of <60 ml/min/1.73m^2^. The median UAE was 6.6 mg/24h, and 8.8% of the total study population had a UAE of ≥30 mg/24h. The accelerometer was worn on average 6.3±1.2 days (95.7% of the participants provided at least 4 valid days of data, 57.5% of the participants provided 7 valid days of data). Average waking time was 15.7±0.9 h/day, of which most time was spent sedentary (9.4±1.7h), and only 2.0±0.7h was spent being physically active. The amount of LPA was on average 1.6±0.6 h/day, and the median HPA was 18.4 [9.0–31.4] min/day. Fig 1 shows the daily percentages of sedentary time, standing time and time spent physically active (stepping) according to eGFR_crcys_ and albuminuria categories. Participants with a lower eGFR_crcys_ and those with a higher UAE spent more time sedentary, so less time standing and physically active in comparison with those with a higher eGFR_crcys_ and those with a lower UAE. Sedentary time was interrupted on average 37.4±8.5 times per day (sedentary breaks). The daily number of prolonged sedentary bouts (accumulated in a consecutive period ≥30 min) was on average 4.9±1.6, and the median average sedentary bout duration was 10.7 [8.7–13.2] min (Table 1).

**Table 1:** Characteristics of the total study population and stratified according to quartiles of total amount of sedentary time.

		Sex-specific quartiles of total amount of sedentary time			
	Total (n = 2258)	Q1 (n = 563)	Q2 (n = 565)	Q3 (n = 565)	Q4 (n = 565)
Range of sedentary time (h/day) in men	4.3–15.9	4.3–8.9	8.9–10.0	10.0–11.0	11.0–15.9
Range of sedentary time (h/day) in women	2.5–14.4	2.5–7.8	7.8–8.8	8.8–9.9	9.9–14.4
Age (years)	60.1 ±8.1	58.8 ±8.3	61.0 ±7.7	60.6 ±8.2	60.2 ±8.0
Men	1159 (51.3)	289 (51.3)	290 (51.3)	290 (51.3)	290 (51.3)
Educational level					
Low	370 (16.4)	97 (17.2)	96 (17.0)	77 (13.6)	100 (17.7)
Intermediate	971 (43.0)	264 (46.9)	245 (43.4)	235 (41.6)	227 (40.2)
High	917 (40.6)	202 (35.9)	224 (39.6)	253 (44.8)	238 (42.1)
Smoking behavior (% current)	281 (12.4)	61 (10.8)	54 (9.6)	58 (10.3)	108 (19.1)
Alcohol consumption (% high)	581 (25.7)	135 (24.0)	151 (26.7)	159 (28.1)	136 (24.1)
Energy intake (kcal/day)	2170 ±602	2217 ±608	2153 ±586	2177 ±593	2136 ±619
Body mass index^a^	27.0 ±4.5	26.1 ±4.3	26.7 ±4.2	26.9 ±4.1	28.4 ±5.0
Waist circumference (cm)					
Men	101.7 ±12.0	97.7 ±11.1	100.5 ±11.0	101.9 ±11.2	106.7 ±12.9
Women	89.5 ±12.6	86.3 ±11.6	87.8 ±11.0	89.3 ±11.2	94.4 ±14.8
Office systolic blood pressure (mmHg)	135.2 ±18.2	134.7 ±18.7	135.3 ±18.3	134.8 ±17.3	136.3 ±18.7
Office diastolic blood pressure (mmHg)	76.2 ±9.9	76.1 ±10.1	75.9 ±10.0	75.9 ±9.3	77.1 ±10.2
Hypertension	1298 (57.5)	275 (48.8)	333 (58.9)	323 (57.2)	367 (65.0)
Antihypertensive medication	920 (40.7)	175 (31.1)	211 (37.3)	235 (41.6)	299 (52.9)
Glucose metabolism status					
Normal glucose metabolism	1268 (56.2)	363 (64.5)	345 (61.1)	320 (56.6)	240 (42.5)
Prediabetes	351 (15.5)	91 (16.2)	81 (14.3)	87 (15.4)	92 (16.3)
Type 2 diabetes	639 (28.3)	109 (19.4)	139 (24.6)	158 (28.0)	233 (41.2)
Total cholesterol (mmol/l)	5.2 ±1.2	5.4 ±1.1	5.3 ±1.2	5.3 ±1.2	5.1 ±1.2
HDL cholesterol (mmol/l)					
Men	1.4 ±0.4	1.5 ±0.4	1.4 ±0.4	1.3 ±0.4	1.2 ±0.3
Women	1.8 ±0.5	1.9 ±0.5	1.9 ±0.5	1.7 ±0.5	1.6 ±0.5
LDL cholesterol (mmol/l)	3.0 ±1.0	3.1 ±1.0	3.1 ±1.0	3.1 ±1.1	2.9 ±1.1
Triglycerides (mmol/l)	1.23 [0.89–1.73]	1.09 [0.83–1.51]	1.19 [0.87–1.58]	1.26 [0.91–1.75]	1.40 [1.01–1.97]
Total-to-HDL cholesterol ratio	3.6 ±1.1	3.5 ±1.1	3.5 ±1.1	3.6 ±1.1	3.8 ±1.2
HbA1c (%)^b^					
No type 2 diabetes	5.5 ±0.4	5.5 ±0.4	5.5 ±0.4	5.5 ±0.4	5.5 ±0.4
Type 2 diabetes	6.9 ±1.0	6.8 ±1.0	7.0 ±1.0	6.8 ±0.9	7.0 ±1.1
Lipid-modifying medication	837 (37.1)	169 (30.0)	192 (34.0)	214 (37.9)	262 (46.4)
Prevalent cardiovascular disease	378 (16.7)	79 (14.0)	93 (16.5)	94 (16.6)	112 (19.8)
Noncardiovascular comorbidity (% with comorbidity)	292 (12.9)	65 (11.5)	65 (11.5)	64 (11.3)	98 (17.3)
Limited mobility (% limited)	367 (16.3)	67 (11.9)	73 (12.9)	82 (14.5)	145 (25.7)
eGFR_crcys_ (ml/min/1.73m^2^)	87.9 ±14.6	90.8 ±13.8	87.8 ±13.7	87.0 ±15.0	86.1 ±15.7
eGFR_crcys_ categories					
≥90 ml/min/1.73m^2^	1078 (47.7)	308 (54.7)	265 (46.9)	256 (45.3)	249 (44.1)
60-<90 ml/min/1.73m^2^	1082 (47.9)	243 (43.2)	281 (49.7)	279 (49.4)	279 (49.4)
<60 ml/min/1.73m^2^	98 (4.3)	12 (2.1)	19 (3.4)	30 (5.3)	37 (6.5)
Urinary albumin excretion rate (mg/24h)	6.6 [3.9–12.3]	6.0 [3.8–11.0]	6.3 [3.7–11.2]	6.6 [3.9–12.6]	7.9 [4.2–15.8]
Urinary albumin excretion categories					
<15 mg/24h	1812 (80.2)	479 (85.1)	465 (82.3)	458 (81.1)	410 (72.6)
15-<30 mg/24h	247 (10.9)	50 (8.9)	55 (9.7)	62 (11.0)	80 (14.2)
≥30 mg/24h	199 (8.8)	34 (6.0)	45 (8.0)	45 (8.0)	75 (13.3)
Valid days (#)	6.3 ±1.2	6.2 ±1.3	6.4 ±1.0	6.4 ±1.0	6.1 ±1.2
Waking time (h/day)	15.7 ±0.9	15.6 ±0.9	15.6 ±0.9	15.7 ±0.9	16.1 ±0.9
Sedentary time (h/day)	9.4 ±1.7	7.4 ±0.9	8.9 ±0.6	9.9 ±0.6	11.4 ±0.9
Standing time (h/day)	4.3 ±1.3	5.6 ±1.2	4.5 ±1.0	3.9 ±0.9	3.2 ±0.8
Total physical activity (h/day)	2.0 ±0.7	2.5 ±0.6	2.1 ±0.6	1.8 ±0.5	1.5 ±0.5
Lower intensity physical activity (h/day)	1.6 ±0.6	2.0 ±0.5	1.7 ±0.5	1.5 ±0.4	1.2 ±0.4
Higher intensity physical activity (min/day)	18.4 [9.0–31.4]	25.3 [14.3–39.6]	19.9 [11.0–31.8]	16.8 [8.1–29.0]	12.4 [5.3–23.0]
Sedentary breaks (#/day)	37.4 ±8.5	38.3 ±9.6	38.1 ±8.3	37.4 ±7.7	35.8 ±8.2
Prolonged sedentary bouts (#/day)	4.9 ±1.6	3.4 ±1.0	4.5 ±1.0	5.2 ±1.1	6.4 ±1.3
Average sedentary bout duration (min)	10.7 [8.7–13.2]	8.7 [7.2–10.3]	10.1 [8.5–12.1]	11.2 [9.6–13.4]	13.2 [11.0–15.7]

*Note*: Data are presented as n (%), mean ±standard deviation, median [interquartile range] or range (only for range of sedentary time).

Abbreviations: eGFR_crcys_, estimated glomerular filtration rate based on serum creatinine and serum cystatin C; HbA1c, hemoglobin A1c (glycated hemoglobin); HDL cholesterol, high-density lipoprotein cholesterol; LDL cholesterol, low-density lipoprotein cholesterol.

^a^ Body mass index was available in n = 2257.

^b^ HbA1c was available in n = 2254.

### Daily activity and eGFR

Table 2 presents the adjusted means of the physical activity and sedentary behavior variables according to eGFR_crcys_ categories. Table 3 presents the associations of these variables with eGFR_crcys_. More daily total physical activity and LPA were associated with a higher eGFR_crcys_ after adjustment for confounders (B_total_ = 2.30 (95%CI = 1.46; 3.14) ml/min/1.73m^2^ per 1 h daily total physical activity; B_LPA_ = 2.10 (1.08; 3.12) ml/min/1.73m^2^ per 1 h daily LPA; model 1). After additional adjustment for potential mediators (models 4), the effect sizes were approximately 30% smaller. More daily HPA was also associated with a higher eGFR_crcys_ after adjustment for confounders and sedentary time (B = 0.53 (0.21; 0.85) ml/min/1.73m^2^ per 10 min daily HPA), but after further adjustment for potential mediators in model 4, the effect size became approximately 40% smaller and the 95% confidence interval included zero (B = 0.31 (-0.02; 0.64) ml/min/1.73m^2^ per 10 min daily HPA). More daily sedentary time was associated with a lower eGFR_crcys_ (B = -0.71 (-1.08; -0.35) ml/min/1.73m^2^ per h daily sedentary time), after adjustment for confounders including HPA (model 2). After additional adjustment for potential mediators (model 4), the effect size became approximately 30% smaller. A larger number of daily sedentary breaks was associated with a higher eGFR_crcys_ in model 2 (B = 0.80 (0.12; 1.47) ml/min/1.73m^2^ per 10 daily sedentary breaks), but after additional adjustment for sedentary time in model 3, the effect size was smaller and the 95% confidence interval included zero (B = 0.59 (-0.10; 1.27) ml/min/1.73m^2^ per 10 daily sedentary breaks). Having more daily prolonged sedentary bouts and having a longer average sedentary bout duration were both associated with a lower eGFR_crcys_, even after adjustment for confounders and potential mediators in models 3 and 4 (B_prolonged bouts_ = -0.57 (-1.14; -0.01) ml/min/1.73m^2^ per daily prolonged sedentary bout; B_bout duration_ = -0.23 (-0.41; -0.04) ml/min/1.73m^2^ per minute of average sedentary bout duration).

**Table 2:** Adjusted means of physical activity and sedentary behavior variables according to eGFR_crcys_ categories.

		≥90 ml/min/1.73m^2^ (n = 1078) Mean (95%CI)	60-<90 ml/min/1.73m^2^ (n = 1082) Mean (95%CI)	<60 ml/min/1.73m^2^ (n = 98) Mean (95%CI)	P value Overall
Total physical activity (h/day)	Model 1	2.05 (2.01; 2.09)	1.94 (1.91; 1.98)	1.82 (1.69; 1.94)	< 0.001
	Model 2	N/A	N/A	N/A	N/A
	Model 3	N/A	N/A	N/A	N/A
	Model 4	2.02 (1.99; 2.06)	1.96 (1.92; 2.00)	1.87 (1.75; 2.00)	0.022
Lower intensity physical activity (h/day)	Model 1	1.65 (1.61; 1.68)	1.58 (1.55; 1.61)	1.50 (1.40; 1.61)	0.006
	Model 2	N/A	N/A	N/A	N/A
	Model 3	N/A	N/A	N/A	N/A
	Model 4	1.63 (1.60; 1.67)	1.59 (1.56; 1.62)	1.53 (1.43; 1.64)	0.087
Higher intensity physical activity (min/day)	Model 1	20.49 (19.59; 21.42)	18.58 (17.72; 19.44)	15.58 (13.08; 18.30)	0.001
	Model 2	N/A	N/A	N/A	N/A
	Model 3	20.17 (19.31; 21.05)	18.79 (17.98; 19.62)	16.51 (14.04; 19.18)	0.014
	Model 4	19.79 (18.96; 20.66)	19.07 (18.26; 19.90)	17.39 (14.87; 20.11)	0.203
Sedentary time (h/day)	Model 1	9.31 (9.21; 9.40)	9.50 (9.41; 9.59)	9.78 (9.48; 10.08)	0.002
	Model 2	9.34 (9.25; 9.43)	9.47 (9.39; 9.56)	9.66 (9.37; 9.95)	0.045
	Model 3	N/A	N/A	N/A	N/A
	Model 4	9.38 (9.29; 9.46)	9.45 (9.36; 9.53)	9.57 (9.28; 9.86)	0.351
Sedentary breaks (#/day)	Model 1	37.99 (37.50; 38.47)	36.83 (36.35; 37.31)	37.46 (35.87; 39.06)	0.006
	Model 2	37.92 (37.43; 38.40)	36.88 (36.40; 37.36)	37.70 (36.11; 39.28)	0.013
	Model 3	37.85 (37.37; 38.33)	36.93 (36.46; 37.40)	37.91 (36.35; 39.48)	0.025
	Model 4	37.82 (37.34; 38.30)	36.95 (36.48; 37.42)	37.99 (36.41; 39.56)	0.034
Prolonged sedentary bouts (#/day)	Model 1	4.70 (4.61; 4.79)	4.97 (4.88; 5.06)	5.24 (4.94; 5.54)	< 0.001
	Model 2	4.73 (4.64; 4.82)	4.95 (4.87; 5.04)	5.13 (4.84; 5.42)	0.001
	Model 3	4.79 (4.73; 4.84)	4.91 (4.85; 4.97)	4.95 (4.76; 5.14)	0.010
	Model 4	4.79 (4.73; 4.85)	4.91 (4.85; 4.97)	4.93 (4.74; 5.12)	0.018
Sedentary bout duration (min)	Model 1	10.38 (10.20; 10.57)	11.01 (10.83; 11.21)	11.27 (10.62; 11.95)	< 0.001
	Model 2	10.44 (10.26; 10.62)	10.98 (10.78; 11.17)	11.05 (10.43; 11.69)	< 0.001
	Model 3	10.53 (10.37; 10.69)	10.91 (10.75; 11.08)	10.78 (10.27; 11.32)	0.005
	Model 4	10.54 (10.38; 10.70)	10.90 (10.74; 11.06)	10.73 (10.22; 11.27)	0.011

*Note*: Means represent adjusted means of the dependent variables in each eGFR_crcys_ category. The means in models 1 were adjusted for age, sex, glucose metabolism status, waking time, educational level, smoking behavior, alcohol consumption, energy intake, comorbid disease, mobility limitation; in models 2 the means of the sedentary behavior variables were additionally adjusted for HPA; in models 3 the means of HPA and the sedentary behavior pattern variables were additionally adjusted for sedentary time; in models 4 all means were additionally adjusted for office systolic blood pressure, use of antihypertensive medication, waist circumference, total-to-HDL cholesterol ratio, triglycerides, use of lipid-modifying medication, prevalent cardiovascular disease. All analyses were based on complete cases (n = 2,258).

Abbreviations: CI, confidence interval; eGFR_crcys_, estimated glomerular filtration rate based on serum creatinine and serum cystatin C; HPA, higher intensity physical activity; HDL cholesterol, high-density lipoprotein cholesterol, N/A, not applicable.

**Table 3:** Associations of physical activity and sedentary behavior variables with eGFR_crcys_.

	Model 1 Beta (95%CI)	Model 2 Beta (95%CI)	Model 3 Beta (95%CI)	Model 4 Beta (95%CI)
Total physical activity (h/day)	**2.30 (1.46; 3.14)**	N/A	N/A	**1.55 (0.69; 2.40)**
Lower intensity physical activity (h/day)	**2.10 (1.08; 3.12)**	N/A	N/A	**1.49 (0.47; 2.50)**
Higher intensity physical activity (10 min/day)	**0.70 (0.39; 1.02)**	N/A	**0.53 (0.21; 0.85)**	0.31 (-0.02; 0.64)
Sedentary time (h/day)	**-0.88 (-1.23; -0.53)**	**-0.71 (-1.08; -0.35)**	N/A	**-0.47 (-0.84; -0.10)**
Sedentary breaks (10/day)	**0.93 (0.26; 1.61)**	**0.80 (0.12; 1.47)**	0.59 (-0.10; 1.27)	0.51 (-0.17; 1.19)
Prolonged sedentary bouts (#/day)	**-0.96 (-1.32; -0.61)**	**-0.82 (-1.19; -0.46)**	**-0.66 (-1.23; -0.09)**	**-0.57 (-1.14; -0.01)**
Average sedentary bout duration (min)	**-0.41 (-0.57; -0.26)**	**-0.35 (-0.51; -0.20)**	**-0.27 (-0.45–0.08)**	**-0.23 (-0.41; -0.04)**

*Note*: Betas represent the difference in eGFR_crcys_ per one unit increase in the independent variable. Boldface indicates statistical significance (P <0.05). The associations in models 1 were adjusted for age, sex, glucose metabolism status, waking time, educational level, smoking behavior, alcohol consumption, energy intake, comorbid disease, mobility limitation; in models 2 the associations with the sedentary behavior variables were additionally adjusted for HPA; in models 3 the associations with HPA and the sedentary behavior pattern variables were additionally adjusted for sedentary time; in models 4 all associations were additionally adjusted for office systolic blood pressure, use of antihypertensive medication, waist circumference, total-to-HDL cholesterol ratio, triglycerides, use of lipid-modifying medication, prevalent cardiovascular disease. All analyses were based on complete cases (n = 2,258).

Abbreviations: CI, confidence interval; eGFR_crcys_, estimated glomerular filtration rate based on serum creatinine and serum cystatin C; HPA, higher intensity physical activity; HDL cholesterol, high-density lipoprotein cholesterol, N/A, not applicable.

### Daily activity and albuminuria

Table 4 presents the adjusted means of the physical activity and sedentary behavior variables according to albuminuria categories. Table 5 presents the associations of these variables with albuminuria. More daily total physical activity was associated with lower ORs for higher UAE, although this was not evident for all quartiles. After adjustment for potential mediators in model 4, effect sizes were largely similar, particularly for a UAE of 15-<30 mg/24h. LPA and HPA were not associated with albuminuria after adjustment for confounders (models 1 and 3). In contrast, more daily sedentary time was associated with a higher odds for a UAE of 15-<30 mg/24h (OR_15-<30mg/24h_ = 1.11 (95%CI = 1.01; 1.22)) and a UAE of ≥30 mg/24h (OR_≥30mg/24h_ = 1.10 (0.99; 1.22)) in model 2. After adjustment for potential mediators in model 4, the strength of the association with a UAE of 15-<30 mg/24h was similar to the association in model 2 (OR_15-<30mg/24h_ = 1.10 (1.00; 1.22)), but the association with a UAE ≥30 mg/24h decreased with approximately 35% (OR_≥30mg/24h_ = 1.06 (0.95; 1.19)). The daily number of sedentary breaks was not associated with albuminuria in any model. Having more daily prolonged sedentary bouts and having a longer average sedentary bout duration were only associated with a higher odds for a UAE of 15-<30 mg/24h and ≥30 mg/24h in models 1.

**Table 4:** Adjusted means of physical activity and sedentary behavior variables according to albuminuria categories.

		<15 mg/24h (n = 1812) Mean (95%CI)	15-<30 mg/24h (n = 247) Mean (95%CI)	≥30 mg/24h (n = 199) Mean (95%CI)	P value Overall
Total physical activity (h/day)	Model 1	2.00 (1.98; 2.03)	1.94 (1.87; 2.02)	1.89 (1.80; 1.98)	0.033
	Model 2	N/A	N/A	N/A	N/A
	Model 3	N/A	N/A	N/A	N/A
	Model 4	2.00 (1.97; 2.03)	1.95 (1.87; 2.03)	1.92 (1.83; 2.01)	0.177
Lower intensity physical activity (h/day)	Model 1	1.62 (1.60; 1.65)	1.57 (1.51; 1.64)	1.54 (1.46; 1.61)	0.062
	Model 2	N/A	N/A	N/A	N/A
	Model 3	N/A	N/A	N/A	N/A
	Model 4	1.62 (1.60; 1.64)	1.58 (1.51; 1.64)	1.55 (1.48; 1.63)	0.173
Higher intensity physical activity (min/day)	Model 1	19.64 (18.97; 20.31)	18.85 (17.11; 20.68)	17.29 (15.41; 19.28)	0.087
	Model 2	N/A	N/A	N/A	N/A
	Model 3	19.50 (18.87; 20.14)	19.36 (17.67; 21.12)	17.89 (16.06; 19.83)	0.306
	Model 4	18.47 (18.81; 20.05)	19.41 (17.75; 21.15)	18.47 (16.62; 20.42)	0.653
Sedentary time (h/day)	Model 1	9.37 (9.30; 9.44)	9.60 (9.41; 9.79)	9.64 (9.43; 9.85)	0.012
	Model 2	9.38 (9.31; 9.45)	9.58 (9.40; 9.76)	9.57 (9.37; 9.78)	0.041
	Model 3	N/A	N/A	N/A	N/A
	Model 4	9.39 (9.32; 9.45)	9.58 (9.40; 9.75)	9.53 (9.33; 9.73)	0.090
Sedentary breaks (#/day)	Model 1	37.56 (37.20; 37.93)	37.06 (36.08; 38.05)	36.45 (35.33; 37.58)	0.156
	Model 2	37.54 (37.18; 37.91)	37.09 (36.11; 38.08)	36.58 (35.46; 37.70)	0.238
	Model 3	37.51 (37.15; 37.86)	37.24 (36.27; 38.21)	36.72 (35.62; 37.83)	0.405
	Model 4	37.48 (37.12; 37.83)	37.31 (36.35; 38.28)	36.91 (35.80; 38.02)	0.637
Prolonged sedentary bouts (#/day)	Model 1	4.81 (4.74; 4.88)	5.01 (4.82; 5.20)	5.08 (4.87; 5.29)	0.015
	Model 2	4.82 (4.75; 4.88)	5.00 (4.82; 5.18)	5.03 (4.82; 5.23)	0.047
	Model 3	4.85 (4.80; 4.89)	4.87 (4.76; 4.99)	4.91 (4.78; 5.04)	0.652
	Model 4	4.85 (4.81; 4.89)	4.87 (4.75; 4.99)	4.89 (4.75; 5.02)	0.835
Sedentary bout duration (min)	Model 1	10.61 (10.48; 10.76)	11.06 (10.65; 11.46)	11.27 (10.82; 11.75)	0.008
	Model 2	10.63 (10.50; 10.77)	11.02 (10.64; 11.43)	11.16 (10.71; 11.61)	0.029
	Model 3	10.68 (10.56; 10.79)	10.84 (10.52; 11.18)	10.98 (10.60; 11.36)	0.263
	Model 4	10.69 (10.60; 10.82)	10.80 (10.49; 11.13)	10.87 (10.50; 11.26)	0.604

*Note*: Means represent adjusted means of the dependent variables in each albuminuria category. The means in models 1 were adjusted for age, sex, glucose metabolism status, waking time, educational level, smoking behavior, alcohol consumption, energy intake, comorbid disease, mobility limitation; in models 2 the means of the sedentary behavior variables were additionally adjusted for HPA; in models 3, the means of HPA and the sedentary behavior pattern variables were additionally adjusted for sedentary time; in models 4 all means were additionally adjusted for office systolic blood pressure, use of antihypertensive medication, waist circumference, total-to-HDL cholesterol ratio, triglycerides, use of lipid-modifying medication, prevalent cardiovascular disease.

All analyses were based on complete cases (n = 2,258).

Abbreviations: CI, confidence interval; eGFR_crcys_, estimated glomerular filtration rate based on serum creatinine and serum cystatin C; HPA, higher intensity physical activity; HDL cholesterol, high-density lipoprotein cholesterol; N/A, not applicable.

**Table 5:** Associations of physical activity and sedentary behavior variables with albuminuria.

			Model 1 OR (95%CI)	Model 2 OR (95%CI)	Model 3 OR (95%CI)	Model 4 OR (95%CI)
Total physical activity (h/day)	<15 mg/24h		Reference	N/A	N/A	Reference
	15-<30 mg/24h	Q1	Reference			Reference
		Q2	**0.59 (0.40; 0.87)**			**0.59 (0.40; 0.88)**
		Q3	**0.67 (0.45; 0.99)**			0.67 (0.45; 1.00)
		Q4	**0.63 (0.41; 0.96)**			0.65 (0.42; 1.00)
	≥30 mg/24h	Q1	Reference			Reference
		Q2	0.75 (0.49; 1.15)			0.83 (0.53; 1.28)
		Q3	**0.59 (0.37; 0.95)**			0.68 (0.42; 1.11)
		Q4	0.84 (0.53; 1.35)			1.06 (0.64; 1.73)
Lower intensity physical activity (h/day)	<15 mg/24h		Reference	N/A	N/A	Reference
	15-<30 mg/24h	Q1	Reference			Reference
		Q2	1.01 (0.70; 1.48)			1.03 (0.70; 1.50)
		Q3	0.80 (0.54; 1.19)			0.81 (0.54; 1.21)
		Q4	0.82 (0.54; 1.24)			0.85 (0.56; 1.29)
	≥30 mg/24h	Q1	Reference			Reference
		Q2	1.00 (0.65; 1.52)			1.08 (0.70; 1.67)
		Q3	**0.54 (0.33; 0.88)**			0.61 (0.37; 1.01)
		Q4	1.02 (0.65; 1.59)			1.21 (0.77; 1.92)
Higher intensity physical activity (10 min/day)	<15 mg/24h		Reference	N/A	Reference	Reference
	15-<30 mg/24h		0.97 (0.89; 1.06)		1.00 (0.91; 1.09)	1.00 (0.91; 1.10)
	≥30 mg/24h		0.91 (0.82; 1.02)		0.94 (0.83; 1.05)	0.96 (0.85; 1.08)
Sedentary time (h/day)	<15 mg/24h		Reference	Reference	N/A	Reference
	15-<30 mg/24h		**1.11 (1.01; 1.22)**	**1.11 (1.01; 1.22)**		1.10 (1.00; 1.22)
	≥30 mg/24h		**1.11 (1.01; 1.24)**	1.10 (0.99; 1.22)		1.06 (0.95; 1.19)
Sedentary breaks (10/day)	<15 mg/24h		Reference	Reference	Reference	Reference
	15-<30 mg/24h		0.92 (0.77; 1.10)	0.93 (0.78; 1.11)	0.96 (0.80; 1.15)	0.97 (0.81; 1.16)
	≥30 mg/24h		0.85 (0.69; 1.04)	0.86 (0.70; 1.05)	0.89 (0.72; 1.09)	0.91 (0.74; 1.12)
Prolonged sedentary bouts (#/day)	<15 mg/24h		Reference	Reference	Reference	Reference
	15-<30 mg/24h		1.10 (1.00; 1.20)	1.09 (0.99; 1.20)	1.03 (0.89; 1.19)	1.02 (0.88; 1.18)
	≥30 mg/24h		**1.11 (1.01; 1.23)**	1.10 (0.99; 1.22)	1.06 (0.91; 1.25)	1.03 (0.88; 1.22)
Average sedentary bout duration (min)	<15 mg/24h		Reference	Reference	Reference	Reference
	15-<30 mg/24h		**1.04 (1.00; 1.08)**	**1.04 (1.00; 1.08)**	1.03 (0.98; 1.07)	1.02 (0.98; 1.07)
	≥30 mg/24h		**1.05 (1.01; 1.09)**	**1.04 (1.00; 1.09)**	1.03 (0.99; 1.09)	1.02 (0.97; 1.07)

*Note*: The odds ratios (OR) represent the odds of having a urinary albumin excretion of 15-<30 mg/24h or a urinary albumin excretion of ≥30 mg/24h (with a urinary albumin excretion of <15 mg/24h as reference category), respectively, relative to the odds in the first quartile for total physical activity and LPA, or per one unit increase in HPA or the sedentary behavior variables. Boldface indicates statistical significance (P <0.05). The associations in models 1 were adjusted for age, sex, glucose metabolism status, waking time, educational level, smoking behavior, alcohol consumption, energy intake, comorbid disease, mobility limitation; in models 2 the associations of the sedentary behavior variables were additionally adjusted for HPA; in models 3 the associations of HPA and the sedentary behavior pattern variables were additionally adjusted for sedentary time; in models 4 all associations were additionally adjusted for office systolic blood pressure, use of antihypertensive medication, waist circumference, total-to-HDL cholesterol ratio, triglycerides, use of lipid-modifying medication, prevalent cardiovascular disease. All analyses were based on complete cases (n = 2,258). Distribution of participants according to albuminuria categories: <15 mg/24h n = 1812, 15-<30 mg/24h n = 247, ≥30 mg/24h n = 199.

Abbreviations: CI, confidence interval; eGFR_crcys_, estimated glomerular filtration rate based on serum creatinine and serum cystatin C; HPA, higher intensity physical activity; HDL cholesterol, high-density lipoprotein cholesterol; N/A, not applicable.

### Additional analyses

In additional analyses, office systolic blood pressure was replaced with 24h average ambulatory systolic blood pressure (n = 1,996). The effect sizes of the associations of all variables with eGFR_crcys_ were somewhat smaller in models 4 and the 95% confidence interval included zero for sedentary time, prolonged sedentary bouts and average sedentary bout duration (S1 Table). However, these analyses were hampered by a loss of statistical power. The effect sizes of the associations of all physical activity and sedentary behavior variables with albuminuria were largely similar (S2 Table). Further, results were similar when participants with <4 valid days (≥10h of waking data) were excluded (S3 and S4 Tables, n = 2,162). Glucose metabolism status may not only be a confounder (to control for the oversampling of individuals with T2DM in The Maastricht Study), but may be a mediator of the associations of physical activity and sedentary behavior with kidney function and kidney damage as well. When models 1 were not adjusted for glucose metabolism status, results were similar for eGFR_crcys_, whereas most associations of albuminuria were somewhat stronger (S5 and S6 Tables, n = 2,258). Further, results were similar when waist circumference was replaced with body mass index (S7 and S8 Tables, n = 2,257). Finally, we explored whether there was statistical interaction between sedentary time and HPA. These analyses showed that the association of sedentary time with eGFR was weaker at higher levels of HPA (*P* value of the interaction term between sedentary time and HPA (*P*_interaction_) 0.021 in model 2). We observed no interaction for the association of sedentary time with albuminuria (*P*_interaction_ 0.283 for UAE_15-<30mg/24h_ and 0.644 for UAE_≥30mg/24h_ in model 2).

## Discussion

To our knowledge, this is the first study in which posture-based accelerometry data were used to measure total amount and patterns of physical activity and sedentary behavior in order to examine associations of the total daily activity spectrum with kidney function and kidney damage. The results demonstrated that physical activity and sedentary behavior were associated with eGFR_crcys_ and albuminuria. More physical activity was associated with a more favorable kidney function and less kidney damage, more sedentary behavior was associated with a more adverse kidney function and more kidney damage. Importantly, additional analyses suggested that higher levels of HPA can somewhat attenuate (*i*.*e*., counterbalance) the adverse association of more sedentary time with kidney function. Positive effects of HPA on health and on kidney function in particular, have been described by others [10,11,13,14] and in this paper, which supports the assumption. In addition, sedentary patterns, expressed by having more sedentary bouts (of at least 30 min) or having a longer average sedentary bout duration, were associated with a more adverse eGFR, even after adjustment for the amount of HPA and sedentary time. This suggests that uninterrupted sitting periods of 30 minutes or longer could negatively affect kidney function.

Remarkably, more total physical activity as well as more LPA and HPA were associated with a more favorable kidney function, whereas only more total physical activity was associated with less kidney damage. The small numbers of participants in the quartiles of LPA may have contributed to these findings. Alternatively, the amounts of LPA and HPA may have been insufficient to detect an association with albuminuria. Also, sedentary patterns were not associated with kidney damage. Since more total sedentary time was associated with more kidney damage, it could be suggested that the total amount of sedentary time may be more important for kidney damage than the way in which it is accumulated. Additionally, even when effect sizes are small, the potential impact on population level may still be relevant as sedentary behavior is highly prevalent on both an inter- and intra-individual level: the majority of individuals has been shown to spend on average more than half of the waking day being sedentary [5–8].

From a pathophysiological perspective, albuminuria is hypothesized to be a biomarker of generalized endothelial dysfunction (*i*.*e*., endothelial dysfunction in the micro- and macrocirculation) [27] and capillary rarefaction [28]. Hence, our results suggest that a sedentary lifestyle may contribute to the development of generalized endothelial dysfunction and/or capillary rarefaction. This notion may be supported by previous studies which have shown effects of sedentariness on the endothelium [29,30]. Similarly, associations of physical activity and sedentary behavior with eGFR many not only indicate associations with kidney function but with extrarenal vascular function as well [31].

The associations of physical activity and sedentary behavior with eGFR and albuminuria may be (partly) mediated by traditional risk factors such as T2DM [20,32,33], higher blood pressure [34], adiposity [35–37], and dyslipidemia [9,35–37]. Therefore, the adjustment for glucose metabolism status (to take into account the oversampling of participants with T2DM in The Maastricht Study) could have resulted in overadjustment bias [38]. Analyses without adjustment for glucose metabolism status suggested that the associations with albuminuria may indeed have been somewhat underestimated. Further, the small changes of the regression coefficients of both eGFR_crcys_ and albuminuria after adding potential mediators including waist circumference, blood pressure, lipid profile and prior CVD in models 4 suggested that these risk factors did not fully explain the associations reported. Therefore, physical activity and sedentary behavior may be associated with eGFR_crcys_ and albuminuria via other mechanisms such as low-grade inflammation [39], endothelial dysfunction [29,30,40], reduced activity of the renin-angiotensin system [41], reduced renal sympathetic nerve activity [40], and/or currently unknown effects.

Adjustment for potential mediators may bias the estimate of the magnitude of the associations through collider bias when so-called mediator-outcome confounders are not addressed adequately [42]. For example, when adjusting for the potential mediators blood pressure and cholesterol in the association of physical activity with kidney function, other factors which may influence the potential mediators and outcome, such as adiposity and smoking, may act as mediator-outcome confounders. Therefore, several lifestyle variables which may be mediator-outcome confounders were added simultaneously in models 4 to reduce the risk of collider bias.

In addition, some factors in models 4, such as adiposity may not only act as mediators, but also as confounders. Given the uncertainty over the exact role of some factors which were added in model 4 as confounders or mediators, and the possibility of collider bias, true effect sizes may range from the values reported in the partly adjusted models to those reported in the fully adjusted models.

Previously, studies have reported on associations of physical activity or sedentary behavior with kidney function. Some studies did show associations of physical activity with eGFR [10,12,14] or albuminuria [11,12], while others did not [13,15]. Similar inconsistent results have been reported in studies on sedentary behavior and eGFR [12,16] or albuminuria [12,16]. This inconsistency could have been due to the use of self-reported measures for physical activity and sedentary behavior, which easily could have been subject to recall and reporting bias [43]. Only a few studies have used accelerometry data to examine associations of physical activity and sedentary behavior with kidney function. In line with our results, it has been reported that in individuals with reduced eGFR and/or albuminuria, smaller amounts of LPA and larger amounts of sedentary time were associated with a lower eGFR [17]. In contrast, a small, longitudinal, observational study conducted among individuals with T2DM has reported that changes in total physical activity and sedentary time were associated with changes in serum creatinine, but not with eGFR and UAE [18]. These inconsistencies with our results may have been caused by differences in study design (cross-sectional vs. longitudinal), sample size and study population, but may also be due to differences in the determination of physical activity and sedentary behavior. We used the activPAL accelerometer, which was worn on the thigh for 24 hour per day. This device classifies activity using data on posture in combination with acceleration, so our estimates of sedentary time were more accurate than those in other studies which were based solely on acceleration [44,45].

The use of 24h posture-based accelerometry data and the measurement of total daily activity were major strengths of our study. In addition, we were the first to examine associations of different constructs of sedentary patterns with eGFR and albuminuria. Another strength was the adjustment for an extensive series of potential confounders, including mutual adjustment for HPA and sedentary time, although residual confounding cannot be excluded.

Some limitations should also be mentioned, of which the most important is the cross-sectional nature of our analyses. Therefore, we cannot make strong causal inferences, and reverse causality cannot be excluded. The previously reported association between changes of daily activity levels with changes in serum creatinine [18] supports the existence of an association in the direction from daily activity levels to kidney function in individuals with a mildly to moderately reduced eGFR. However, this study was small. Thus, larger, causal studies are needed to confirm the direction of the association. Another limitation is that, due to missing data, we had to exclude ~1,200 participants. However, the excluded participants did not differ from our study sample with regard to demographic factors, accelerometry variables and kidney function. Further, LPA and HPA were based on step frequency, which is less precise than using acceleration to determine intensity levels. However, it has been demonstrated that a step frequency >~100 steps/minute equals a metabolic equivalent of task (MET) score of ≥3.0 [46] and we used a cut-off point >110 steps/minute for HPA. Finally, our study population consisted of a relatively healthy population of primarily Caucasians from European descent with well-controlled individuals with T2DM. Therefore, the results might not be representative for the general population of adults aged 40–75 years or other ethnicities.

To conclude, this large posture-based accelerometry study showed that both total physical activity and sedentary behavior were associated with eGFR_crcys_ and albuminuria. Additionally, sedentary patterns may be of importance for eGFR_crcys_. Therefore, not only increasing physical activity levels, but also decreasing the amount of sedentary time may prove to be relevant strategies to prevent lower kidney function and kidney damage. However, causal accelerometry studies are required to confirm our results and to further disentangle the associations of (patterns of) daily activity with kidney function and kidney damage.

## Supporting information

S1 Table Associations of physical activity and sedentary behavior variables with eGFR_crcys_ in subpopulation with 24h average ambulatory blood pressure data (n = 1,996). (DOCX)

S2 Table Associations of physical activity and sedentary behavior variables with albuminuria in subpopulation with 24h average ambulatory blood pressure data (n = 1,996). (DOCX)

S3 Table Associations of physical activity and sedentary behaviour variables with eGFR_crcys_ in subpopulation with ≥4 valid days. (DOCX)

S4 Table Associations of physical activity and sedentary behaviour variables with albuminuria in subpopulation with ≥4 valid days. (DOCX)

S5 Table Association of physical activity and sedentary behaviour variables with eGFR_crcys_ with and without adjustment for glucose metabolism status. (DOCX)

S6 Table Association of physical activity and sedentary behaviour variables with albuminuria with and without adjustment for glucose metabolism status. (DOCX)

S7 Table Associations of physical activity and sedentary behavior variables with eGFR_crcys_ adjusted for body mass index instaead of waist circumference (n = 2,257). (DOCX)

S8 Table Associations of physical activity and sedentary behavior variables with albuminuria adjusted for body mass index instead of waist circumference (n = 2,257). (DOCX)

## References

[1] Coresh J, Selvin E, Stevens LA, Manzi J, Kusek JW, Eggers P, et al Prevalence of chronic kidney disease in the United States. Jama. 2007;298(17):2038–47. doi: 10.1001/jama.298.17.2038 . 17986697 10.1001/jama.298.17.2038

[2] Fox CS, Matsushita K, Woodward M, Bilo HJ, Chalmers J, Heerspink HJ, et al Associations of kidney disease measures with mortality and end-stage renal disease in individuals with and without diabetes: a meta-analysis. Lancet. 2012;380(9854):1662–73. doi: 10.1016/S0140-6736(12)61350-6 . 23013602 10.1016/S0140-6736(12)61350-6 PMC3771350

[3] James MT, Hemmelgarn BR, Wiebe N, Pannu N, Manns BJ, Klarenbach SW, et al Glomerular filtration rate, proteinuria, and the incidence and consequences of acute kidney injury: a cohort study. Lancet. 2010;376(9758):2096–103. Epub 2010/11/26. doi: 10.1016/S0140-6736(10)61271-8 . 21094997 10.1016/S0140-6736(10)61271-8

[4] Ainsworth BE, Haskell WL, Whitt MC, Irwin ML, Swartz AM, Strath SJ, et al Compendium of physical activities: an update of activity codes and MET intensities. Med Sci Sports Exerc. 2000;32(9 Suppl):S498–504. Epub 2000/09/19. . 10993420 10.1097/00005768-200009001-00009

[5] Matthews CE, Chen KY, Freedson PS, Buchowski MS, Beech BM, Pate RR, et al Amount of time spent in sedentary behaviors in the United States, 2003–2004. Am J Epidemiol. 2008;167(7):875–81. doi: 10.1093/aje/kwm390 . 18303006 10.1093/aje/kwm390 PMC3527832

[6] WHO: World Health Organizastion. Global status report on noncommunicable diseases 2014. 2014.

[7] Davis MG, Fox KR, Hillsdon M, Sharp DJ, Coulson JC, Thompson JL. Objectively measured physical activity in a diverse sample of older urban UK adults. Med Sci Sports Exerc. 2011;43(4):647–54. doi: 10.1249/MSS.0b013e3181f36196 . 20689449 10.1249/MSS.0b013e3181f36196

[8] Arnardottir NY, Koster A, Van Domelen DR, Brychta RJ, Caserotti P, Eiriksdottir G, et al Objective measurements of daily physical activity patterns and sedentary behaviour in older adults: Age, Gene/Environment Susceptibility-Reykjavik Study. Age and ageing. 2013;42(2):222–9. doi: 10.1093/ageing/afs160 . 23117467 10.1093/ageing/afs160 PMC3575120

[9] Brocklebank LA, Falconer CL, Page AS, Perry R, Cooper AR. Accelerometer-measured sedentary time and cardiometabolic biomarkers: A systematic review. Prev Med. 2015;76:92–102. Epub 2015/04/29. doi: 10.1016/j.ypmed.2015.04.013 . 25913420 10.1016/j.ypmed.2015.04.013

[10] Finkelstein J, Joshi A, Hise MK. Association of physical activity and renal function in subjects with and without metabolic syndrome: a review of the Third National Health and Nutrition Examination Survey (NHANES III). Am J Kidney Dis. 2006;48(3):372–82. Epub 2006/08/26. doi: 10.1053/j.ajkd.2006.05.013 . 16931210 10.1053/j.ajkd.2006.05.013

[11] Robinson ES, Fisher ND, Forman JP, Curhan GC. Physical activity and albuminuria. Am J Epidemiol. 2010;171(5):515–21. Epub 2010/02/06. doi: 10.1093/aje/kwp442 . 20133515 10.1093/aje/kwp442 PMC2842220

[12] Bharakhada N, Yates T, Davies MJ, Wilmot EG, Edwardson C, Henson J, et al Association of sitting time and physical activity with CKD: a cross-sectional study in family practices. Am J Kidney Dis. 2012;60(4):583–90. Epub 2012/06/22. doi: 10.1053/j.ajkd.2012.04.024 . 22717340 10.1053/j.ajkd.2012.04.024

[13] Chang A, Van Horn L, Jacobs DR Jr., Liu K, Muntner P, Newsome B, et al Lifestyle-related factors, obesity, and incident microalbuminuria: the CARDIA (Coronary Artery Risk Development in Young Adults) study. Am J Kidney Dis. 2013;62(2):267–75. Epub 2013/04/23. doi: 10.1053/j.ajkd.2013.02.363 . 23601954 10.1053/j.ajkd.2013.02.363 PMC3720776

[14] Robinson-Cohen C, Littman AJ, Duncan GE, Weiss NS, Sachs MC, Ruzinski J, et al Physical activity and change in estimated GFR among persons with CKD. J Am Soc Nephrol. 2014;25(2):399–406. Epub 2013/12/18. doi: 10.1681/ASN.2013040392 . 24335971 10.1681/ASN.2013040392 PMC3904564

[15] Herber-Gast GC, Hulsegge G, Hartman L, Verschuren WM, Stehouwer CD, Gansevoort RT, et al Physical Activity Is not Associated with Estimated Glomerular Filtration Rate among Young and Middle-Aged Adults: Results from the Population-Based Longitudinal Doetinchem Study. PloS one. 2015;10(10):e0133864 Epub 2015/10/16. doi: 10.1371/journal.pone.0133864 . 26465150 10.1371/journal.pone.0133864 PMC4605681

[16] White SL, Dunstan DW, Polkinghorne KR, Atkins RC, Cass A, Chadban SJ. Physical inactivity and chronic kidney disease in Australian adults: the AusDiab study. Nutr Metab Cardiovasc Dis. 2011;21(2):104–12. Epub 2009/11/27. doi: 10.1016/j.numecd.2009.08.010 . 19939649 10.1016/j.numecd.2009.08.010

[17] Hawkins MS, Sevick MA, Richardson CR, Fried LF, Arena VC, Kriska AM. Association between physical activity and kidney function: National Health and Nutrition Examination Survey. Med Sci Sports Exerc. 2011;43(8):1457–64. Epub 2011/01/05. doi: 10.1249/MSS.0b013e31820c0130 . 21200336 10.1249/MSS.0b013e31820c0130

[18] Guo VY, Brage S, Ekelund U, Griffin SJ, Simmons RK. Objectively measured sedentary time, physical activity and kidney function in people with recently diagnosed Type 2 diabetes: a prospective cohort analysis. Diabet Med. 2015 Epub 2015/08/19. doi: 10.1111/dme.12886 . 26282583 10.1111/dme.12886 PMC5017300

[19] Benatti FB, Ried-Larsen M. The Effects of Breaking up Prolonged Sitting Time: A Review of Experimental Studies. Med Sci Sports Exerc. 2015;47(10):2053–61. Epub 2015/09/18. doi: 10.1249/MSS.0000000000000654 . 26378942 10.1249/MSS.0000000000000654

[20] van der Berg JD, Stehouwer CD, Bosma H, van der Velde JH, Willems PJ, Savelberg HH, et al Associations of total amount and patterns of sedentary behaviour with type 2 diabetes and the metabolic syndrome: The Maastricht Study. Diabetologia. 2016;59(4):709–18. doi: 10.1007/s00125-015-3861-8 . 26831300 10.1007/s00125-015-3861-8 PMC4779127

[21] Schram MT, Sep SJ, van der Kallen CJ, Dagnelie PC, Koster A, Schaper N, et al The Maastricht Study: an extensive phenotyping study on determinants of type 2 diabetes, its complications and its comorbidities. Eur J Epidemiol. 2014;29(6):439–51. Epub 2014/04/24. doi: 10.1007/s10654-014-9889-0 . 24756374 10.1007/s10654-014-9889-0

[22] van der Berg JD, Willems PJ, van der Velde JH, Savelberg HH, Schaper NC, Schram MT, et al Identifying waking time in 24-h accelerometry data in adults using an automated algorithm. J Sports Sci. 2016:1–7. doi: 10.1080/02640414.2016.1140908 . 26837855 10.1080/02640414.2016.1140908

[23] Inker LA, Schmid CH, Tighiouart H, Eckfeldt JH, Feldman HI, Greene T, et al Estimating glomerular filtration rate from serum creatinine and cystatin C. N Engl J Med. 2012;367(1):20–9. doi: 10.1056/NEJMoa1114248 . 22762315 10.1056/NEJMoa1114248 PMC4398023

[24] Martens RJ, Kooman JP, Stehouwer CD, Dagnelie PC, van der Kallen CJ, Koster A, et al Estimated GFR, Albuminuria, and Cognitive Performance: The Maastricht Study. Am J Kidney Dis. 2017;69(2):179–91. Epub 2016/06/14. doi: 10.1053/j.ajkd.2016.04.017 . 27291486 10.1053/j.ajkd.2016.04.017

[25] Altman DG. Practical statistics for medical research. London: Chapman and Hall/CRC; 1990 p. 143–5.

[26] Lee IM, Shiroma EJ, Lobelo F, Puska P, Blair SN, Katzmarzyk PT. Effect of physical inactivity on major non-communicable diseases worldwide: an analysis of burden of disease and life expectancy. Lancet. 2012;380(9838):219–29. Epub 2012/07/24. doi: 10.1016/S0140-6736(12)61031-9 . 22818936 10.1016/S0140-6736(12)61031-9 PMC3645500

[27] Stehouwer CD, Smulders YM. Microalbuminuria and risk for cardiovascular disease: Analysis of potential mechanisms. J Am Soc Nephrol. 2006;17(8):2106–11. doi: 10.1681/ASN.2005121288 . 16825333 10.1681/ASN.2005121288

[28] Martens RJ, Henry RM, Houben AJ, van der Kallen CJ, Kroon AA, Schalkwijk CG, et al Capillary Rarefaction Associates with Albuminuria: The Maastricht Study. J Am Soc Nephrol. 2016;27(12):3748–57. doi: 10.1681/ASN.2015111219 . 27160406 10.1681/ASN.2015111219 PMC5118486

[29] Thosar SS, Bielko SL, Mather KJ, Johnston JD, Wallace JP. Effect of prolonged sitting and breaks in sitting time on endothelial function. Med Sci Sports Exerc. 2015;47(4):843–9. Epub 2014/08/20. doi: 10.1249/MSS.0000000000000479 . 25137367 10.1249/MSS.0000000000000479

[30] Restaino RM, Holwerda SW, Credeur DP, Fadel PJ, Padilla J. Impact of prolonged sitting on lower and upper limb micro- and macrovascular dilator function. Exp Physiol. 2015;100(7):829–38. Epub 2015/05/02. doi: 10.1113/EP085238 . 25929229 10.1113/EP085238 PMC4956484

[31] Tonelli M, Pfeffer MA. Kidney disease and cardiovascular risk. Annual review of medicine. 2007;58:123–39. doi: 10.1146/annurev.med.58.071105.111123 . 17081079 10.1146/annurev.med.58.071105.111123

[32] Wilmot EG, Edwardson CL, Achana FA, Davies MJ, Gorely T, Gray LJ, et al Sedentary time in adults and the association with diabetes, cardiovascular disease and death: systematic review and meta-analysis. Diabetologia. 2012;55(11):2895–905. Epub 2012/08/15. doi: 10.1007/s00125-012-2677-z . 22890825 10.1007/s00125-012-2677-z

[33] Aune D, Norat T, Leitzmann M, Tonstad S, Vatten LJ. Physical activity and the risk of type 2 diabetes: a systematic review and dose-response meta-analysis. Eur J Epidemiol. 2015;30(7):529–42. Epub 2015/06/21. doi: 10.1007/s10654-015-0056-z . 26092138 10.1007/s10654-015-0056-z

[34] Huai P, Xun H, Reilly KH, Wang Y, Ma W, Xi B. Physical activity and risk of hypertension: a meta-analysis of prospective cohort studies. Hypertension. 2013;62(6):1021–6. doi: 10.1161/HYPERTENSIONAHA.113.01965 . 24082054 10.1161/HYPERTENSIONAHA.113.01965

[35] Kelley GA, Kelley KS. Aerobic exercise and lipids and lipoproteins in men: a meta-analysis of randomized controlled trials. J Mens Health Gend. 2006;3(1):61–70. Epub 2008/07/23. . 18645633 10.1016/j.jmhg.2005.09.003 PMC2475654

[36] Kelley GA, Kelley KS, Tran ZV. Aerobic exercise and lipids and lipoproteins in women: a meta-analysis of randomized controlled trials. J Womens Health (Larchmt). 2004;13(10):1148–64. Epub 2005/01/15. doi: 10.1089/jwh.2004.13.1148 . 15650348 10.1089/jwh.2004.13.1148 PMC2447858

[37] Cooper AR, Sebire S, Montgomery AA, Peters TJ, Sharp DJ, Jackson N, et al Sedentary time, breaks in sedentary time and metabolic variables in people with newly diagnosed type 2 diabetes. Diabetologia. 2012;55(3):589–99. Epub 2011/12/15. doi: 10.1007/s00125-011-2408-x . 22167127 10.1007/s00125-011-2408-x

[38] Schisterman EF, Cole SR, Platt RW. Overadjustment bias and unnecessary adjustment in epidemiologic studies. Epidemiology. 2009;20(4):488–95. doi: 10.1097/EDE.0b013e3181a819a1 . 19525685 10.1097/EDE.0b013e3181a819a1 PMC2744485

[39] Henson J, Yates T, Edwardson CL, Khunti K, Talbot D, Gray LJ, et al Sedentary time and markers of chronic low-grade inflammation in a high risk population. PloS one. 2013;8(10):e78350 Epub 2013/11/10. doi: 10.1371/journal.pone.0078350 . 24205208 10.1371/journal.pone.0078350 PMC3812126

[40] Padilla J, Simmons GH, Bender SB, Arce-Esquivel AA, Whyte JJ, Laughlin MH. Vascular effects of exercise: endothelial adaptations beyond active muscle beds. Physiology (Bethesda). 2011;26(3):132–45. Epub 2011/06/15. doi: 10.1152/physiol.00052.2010 . 21670160 10.1152/physiol.00052.2010 PMC3286126

[41] Goessler K, Polito M, Cornelissen VA. Effect of exercise training on the renin-angiotensin-aldosterone system in healthy individuals: a systematic review and meta-analysis. Hypertens Res. 2015 Epub 2015/09/25. doi: 10.1038/hr.2015.100 . 26399454 10.1038/hr.2015.100

[42] Richiardi L, Bellocco R, Zugna D. Mediation analysis in epidemiology: methods, interpretation and bias. Int J Epidemiol. 2013;42(5):1511–9. doi: 10.1093/ije/dyt127 . 24019424 10.1093/ije/dyt127

[43] Atkin AJ, Gorely T, Clemes SA, Yates T, Edwardson C, Brage S, et al Methods of Measurement in epidemiology: sedentary Behaviour. Int J Epidemiol. 2012;41(5):1460–71. Epub 2012/10/10. doi: 10.1093/ije/dys118 . 23045206 10.1093/ije/dys118 PMC3465769

[44] Kozey-Keadle S, Libertine A, Lyden K, Staudenmayer J, Freedson PS. Validation of wearable monitors for assessing sedentary behavior. Med Sci Sports Exerc. 2011;43(8):1561–7. Epub 2011/01/15. doi: 10.1249/MSS.0b013e31820ce174 . 21233777 10.1249/MSS.0b013e31820ce174

[45] Edwardson CL, Rowlands AV, Bunnewell S, Sanders J, Esliger DW, Gorely T, et al Accuracy of Posture Allocation Algorithms for Thigh- and Waist-Worn Accelerometers. Med Sci Sports Exerc. 2016 Epub 2016/01/08. doi: 10.1249/MSS.0000000000000865 . 26741122 10.1249/MSS.0000000000000865

[46] Tudor-Locke C, Craig CL, Brown WJ, Clemes SA, De Cocker K, Giles-Corti B, et al How many steps/day are enough? For adults. Int J Behav Nutr Phys Act. 2011;8:79 Epub 2011/07/30. doi: 10.1186/1479-5868-8-79 . 21798015 10.1186/1479-5868-8-79 PMC3197470

