# Supplementary material for: Amount and pattern of physical activity and sedentary behavior are associated with kidney function and kidney damage: The Maastricht Study
Source: PLoS One. 2018 Apr 4;13(4):e0195306. doi: 10.1371/journal.pone.0195306 (PMC5884554; doi:10.1371/journal.pone.0195306)
Supplement: S1 Table — (DOCX) [file pone.0195306.s001.docx]

S1 Table. Associations of physical activity and sedentary behavior variables with eGFR_crcys_ in subpopulation with 24h average ambulatory blood pressure data (n=1,996)

|  | Model 1  Beta (95%CI) | Model 2  Beta (95%CI) | Model 3  Beta (95%CI) | Model 4  Beta (95%CI) |
| --- | --- | --- | --- | --- |
| Total physical activity (h/day) | **2.20 (1.32; 3.09)** | N/A | N/A | **1.39 (0.49; 2.28)** |
| Lower intensity physical activity (h/day) | **1.98 (0.91; 3.04)** | N/A | N/A | **1.36 (0.30; 2.42)** |
| Higher intensity physical activity (10 min/day) | **0.69 (0.36; 1.02)** | N/A | **0.54 (0.20; 0.88)** | 0.27 (-0.08; 0.61) |
| Sedentary time (h/day) | **-0.79 (-1.17; -0.42)** | **-0.63 (-1.02; -0.24)** | N/A | -0.38 (-0.77; 0.01) |
| Sedentary breaks (10/day) | 0.67 (-0.06; 1.39) | 0.54 (-0.18; 1.26) | 0.36 (-0.37; 1.09) | 0.23 (-0.50; 0.95) |
| Prolonged sedentary bouts (#/day) | **-0.85 (-1.24; -0.47)** | **-0.71 (-1.10; -0.32)** | -0.54 (-1.15; 0.07) | -0.42 (-1.02; 0.19) |
| Average sedentary bout duration (min) | **-0.35 (-0.51; -0.18)** | **-0.29 (-0.46; -0.12)** | **-0.20 (-0.40 -0.01)** | -0.14 (-0.33; 0.06) |

*Note:* Betas represent the difference in eGFR_crcys_ per one unit increase in the independent variable. Boldface indicates statistical significance (P <0.05). The associations in models 1 were adjusted for age, sex, glucose metabolism status, waking time, educational level, smoking behavior, alcohol consumption, energy intake, comorbid disease, mobility limitation; in models 2 the associations with the sedentary behavior variables were additionally adjusted for HPA; in models 3 the associations with HPA and the sedentary behavior pattern variables were additionally adjusted for sedentary time; in models 4 all associations were additionally adjusted for 24h average ambulatory systolic blood pressure, use of antihypertensive medication, waist circumference, total-to-HDL cholesterol ratio, triglycerides, use of lipid-modifying medication, prevalent cardiovascular disease. All analyses were based on complete cases (n=1,996).

Abbreviations: CI, confidence interval; eGFR_crcys_, estimated glomerular filtration rate based on serum creatinine and serum cystatin C; HPA, higher intensity physical activity; HDL cholesterol, high-density lipoprotein cholesterol, N/A, not applicable.
