# Supplementary material for: Amount and pattern of physical activity and sedentary behavior are associated with kidney function and kidney damage: The Maastricht Study
Source: PLoS One. 2018 Apr 4;13(4):e0195306. doi: 10.1371/journal.pone.0195306 (PMC5884554; doi:10.1371/journal.pone.0195306)
Supplement: S3 Table — (DOCX) [file pone.0195306.s003.docx]

S3 Table. Associations of physical activity and sedentary behaviour variables with eGFR_crcys_ in subpopulation with ≥4 valid days (n=2,162)

|  | Model 1  Beta (95%CI) | Model 2  Beta (95%CI) | Model 3  Beta (95%CI) | Model 4  Beta (95%CI) |
| --- | --- | --- | --- | --- |
| Total physical activity (h/day) | **2.33 (1.46; 3.21)** | N/A | N/A | **1.58 (0.70; 2.47)** |
| Lower intensity physical activity (h/day) | **2.11 (1.06; 3.17)** | N/A | N/A | **1.52 (0.48; 2.57)** |
| Higher intensity physical activity (10 min/day) | **0.72 (0.40; 1.04)** | N/A | **0.52 (0.19; 0.86)** | 0.30 (-0.04; 0.63) |
| Sedentary time (h/day) | **-0.96 (-1.33; -0.59)** | **-0.79 (-1.17; -0.41)** | N/A | **-0.56 (-0.94; -0.18)** |
| Sedentary breaks (10/day) | **1.01 (0.30; 1.71)** | **0.86 (0.15; 1.56)** | 0.59 (-0.13; 1.31) | 0.51 (-0.21; 1.22) |
| Prolonged sedentary bouts (#/day) | **-1.03 (-1.40; -0.66)** | **-0.89 (-1.27; -0.51)** | **-0.69 (-1.29; -0.10)** | **-0.60 (-1.19; -0.01)** |
| Average sedentary bout duration (min) | **-0.45 (-0.61; -0.28)** | **-0.38 (-0.55; -0.22)** | **-0.28 (-0.47 -0.08)** | **-0.24 (-0.43; -0.04)** |

*Note:* Betas represent the difference in eGFR_crcys_ per one unit increase in the independent variable. Boldface indicates statistical significance (P <0.05). The associations in models 1 were adjusted for age, sex, glucose metabolism status, waking time, educational level, smoking behavior, alcohol consumption, energy intake, comorbid disease, mobility limitation; in models 2 the associations with the sedentary behavior variables were additionally adjusted for HPA; in models 3 the associations with HPA and the sedentary behavior pattern variables were additionally adjusted for sedentary time; in models 4 all associations were additionally adjusted for office systolic blood pressure, use of antihypertensive medication, waist circumference, total-to-HDL cholesterol ratio, triglycerides, use of lipid-modifying medication, prevalent cardiovascular disease. All analyses were based on complete cases (n=2,162).

Abbreviations: CI, confidence interval; eGFR_crcys_, estimated glomerular filtration rate based on serum creatinine and serum cystatin C; HPA, higher intensity physical activity; HDL cholesterol, high-density lipoprotein cholesterol, N/A, not applicable.
