# Supplementary material for: Amount and pattern of physical activity and sedentary behavior are associated with kidney function and kidney damage: The Maastricht Study
Source: PLoS One. 2018 Apr 4;13(4):e0195306. doi: 10.1371/journal.pone.0195306 (PMC5884554; doi:10.1371/journal.pone.0195306)
Supplement: S5 Table — (DOCX) [file pone.0195306.s005.docx]

S5 Table. Associations of physical activity and sedentary behaviour variables with eGFR_crcys_ with and without adjustment for glucose metabolism status

|  | Model 1  with glucose metabolism status | Model 1  without glucose metabolism status |
| --- | --- | --- |
| Independent variable | Beta (95% CI) | Beta (95% CI) |
| Total physical activity (h/day) | **2.30 (1.46; 3.14)** | **2.27 (1.44; 3.09)** |
| Lower intensity physical activity (h/day) | **2.10 (1.08; 3.12)** | **2.12 (1.11; 3.12)** |
| Higher intensity physical activity (10 min/day) | **0.70 (0.39; 1.02)** | **0.70 (0.39; 1.01)** |
| Sedentary time (h/day) | **-0.88 (-1.23; -0.53)** | **-0.87 (-1.22; -0.53)** |
| Sedentary breaks (10/day) | **0.93 (0.26; 1.61)** | **0.94 (0.27; 1.62)** |
| Prolonged sedentary bouts (#/day) | **-0.96 (-1.32; -0.61)** | **-0.96 (-1.32; -0.61)** |
| Average sedentary bout duration (min) | **-0.41 (-0.57; -0.26)** | **-0.41 (-0.57; -0.26)** |

*Note:* Betas represent the difference in eGFR_crcys_ per one unit increase in the independent variable. Boldface indicates statistical significance (P <0.05). The associations in models 1 were adjusted for age, sex, waking time, educational level, smoking behavior, alcohol consumption, energy intake, comorbid disease, and mobility limitation, with and without additional adjustment for glucose metabolism status. All analyses were based on complete cases (n=2,258).

Abbreviations: CI, confidence interval; eGFR_crcys_, estimated glomerular filtration rate based on serum creatinine and serum cystatin C; HPA, higher intensity physical activity.
