# Supplementary material for: Amount and pattern of physical activity and sedentary behavior are associated with kidney function and kidney damage: The Maastricht Study
Source: PLoS One. 2018 Apr 4;13(4):e0195306. doi: 10.1371/journal.pone.0195306 (PMC5884554; doi:10.1371/journal.pone.0195306)
Supplement: S6 Table — (DOCX) [file pone.0195306.s006.docx]

S6 Table. Associations of physical activity and sedentary behaviour variables with albuminuria with and without adjustment for glucose metabolism status

|  |  |  | Model 1  with glucose metabolism status | Model 1  without glucose metabolism status |
| --- | --- | --- | --- | --- |
|  |  |  | OR (95%CI) | OR (95%CI) |
| Total physical activity (h/day) | <15 mg/24h  15-30 mg/24h  ≥30 mg/24h | Q1  Q2  Q3  Q4  Q1  Q2  Q3  Q4 | Reference  Reference  **0.59 (0.40; 0.87)**  **0.67 (0.45; 0.99)**  **0.63 (0.41; 0.96)**  Reference  0.75 (0.49; 1.15)  **0.59 (0.37; 0.95)**  0.84 (0.53; 1.35) | Reference  Reference  **0.53 (0.36; 0.77)**  **0.58 (0.39; 0.84)**  **0.51 (0.34; 0.76)**  Reference  **0.65 (0.43; 0.98)**  **0.48 (0.31; 0.77)**  **0.62 (0.40; 0.98)** |
| Lower intensity physical activity (h/day) | <15 mg/24h  15-30 mg/24h  ≥30 mg/24h | Q1  Q2  Q3  Q4  Q1  Q2  Q3  Q4 | Reference  Reference  1.01 (0.70; 1.48)  0.80 (0.54; 1.19)  0.82 (0.54; 1.24)  Reference  1.00 (0.65; 1.52)  **0.54 (0.33; 0.88)**  1.02 (0.65; 1.59) | Reference  Reference  0.88 (0.61; 1.27)  0.70 (0.47; 1.03)  0.68 (0.45; 1.01)  Reference  0.82 (0.54; 1.24)  **0.45 (0.28; 0.73)**  0.78 (0.51; 1.20) |
| Higher intensity physical activity (10 min/day) | <15 mg/24h  15-30 mg/24h  ≥30 mg/24h |  | Reference  0.97 (0.89; 1.06)  0.91 (0.82; 1.02) | Reference  0.93 (0.85; 1.01)  **0.86 (0.77; 0.96)** |
| Sedentary time (h/day) | <15 mg/24h  15-30 mg/24h  ≥30 mg/24h |  | Reference  **1.11 (1.01; 1.22)**  **1.11 (1.01; 1.24)** | Reference  **1.17 (1.06; 1.28)**  **1.19 (1.07; 1.32)** |
| Prolonged sedentary bouts (#/day) | <15 mg/24h  15-30 mg/24h  ≥30 mg/24h |  | Reference  1.10 (1.00; 1.20)  **1.11 (1.01; 1.23)** | Reference  **1.13 (1.04; 1.24)**  **1.16 (1.05; 1.28)** |
| Average sedentary bout duration (min) | <15 mg/24h  15-30 mg/24h  ≥30 mg/24h |  | Reference  **1.04 (1.00; 1.08)**  **1.05 (1.01; 1.09)** | Reference  **1.06 (1.02; 1.10)**  **1.07 (1.03; 1.11)** |

*Note:* The odds ratios (OR) represent the odds of having a urinary albumin excretion of 15-<30 mg/24h or a urinary albumin excretion of ≥30 mg/24h (with a urinary albumin excretion of <15 mg/24h as reference category), respectively, relative to the odds in the first quartile for total physical activity and LPA, or per one unit increase in HPA or the sedentary behavior variables. Boldface indicates statistical significance (P <0.05). The associations in models 1 were adjusted for age, sex, waking time, educational level, smoking behavior, alcohol consumption, energy intake, comorbid disease, and mobility limitation, with and without additional adjustment for glucose metabolism status. All analyses were based on complete cases (n=2,258). Distribution of participants according to albuminuria categories: <15 mg/24h n=1812, 15-<30 mg/24h n=247, ≥30 mg/24h n=199.

Abbreviations: CI, confidence interval; eGFR_crcys_, estimated glomerular filtration rate based on serum creatinine and serum cystatin C; HPA, higher intensity physical activity.
