# Supplementary material for: Amount and pattern of physical activity and sedentary behavior are associated with kidney function and kidney damage: The Maastricht Study
Source: PLoS One. 2018 Apr 4;13(4):e0195306. doi: 10.1371/journal.pone.0195306 (PMC5884554; doi:10.1371/journal.pone.0195306)
Supplement: S7 Table — (DOCX) [file pone.0195306.s007.docx]

S7 Table. Associations of physical activity and sedentary behavior variables with eGFR_crcys_ adjusted for body mass index instead of waist circumference (n=2,257)

|  | Model 4  Beta (95%CI) |
| --- | --- |
| Total physical activity (h/day) | **1.55 (0.70; 2.40)** |
| Lower intensity physical activity (h/day) | **1.46 (0.45; 2.48)** |
| Higher intensity physical activity (10 min/day) | 0.32 (-0.01; 0.64) |
| Sedentary time (h/day) | **-0.49 (-0.85; -0.12)** |
| Sedentary breaks (10/day) | 0.49 (-0.19; 1.17) |
| Prolonged sedentary bouts (#/day) | -0.55 (-1.11; 0.01) |
| Average sedentary bout duration (min) | **-0.22 (-0.40; -0.03)** |

*Note:* Betas represent the difference in eGFR_crcys_ per one unit increase in the independent variable. Boldface indicates statistical significance (P <0.05). The associations in models 4 were adjusted for age, sex, glucose metabolism status, waking time, educational level, smoking behavior, alcohol consumption, energy intake, comorbid disease, mobility limitation, HPA (for the sedentary behavior variables only), sedentary time (for HPA and the sedentary behavior pattern variables only), office systolic blood pressure, use of antihypertensive medication, body mass index, total-to-HDL cholesterol ratio, triglycerides, use of lipid-modifying medication, prevalent cardiovascular disease. All analyses were based on complete cases (n=2,257).

Abbreviations: CI, confidence interval; eGFR_crcys_, estimated glomerular filtration rate based on serum creatinine and serum cystatin C; HPA, higher intensity physical activity; HDL cholesterol, high-density lipoprotein cholesterol, N/A, not applicable.
