# Supplementary material for: Amount and pattern of physical activity and sedentary behavior are associated with kidney function and kidney damage: The Maastricht Study
Source: PLoS One. 2018 Apr 4;13(4):e0195306. doi: 10.1371/journal.pone.0195306 (PMC5884554; doi:10.1371/journal.pone.0195306)
Supplement: S8 Table — (DOCX) [file pone.0195306.s008.docx]

S8 Table. Associations of physical activity and sedentary behavior variables with albuminuria adjusted for body mass index instead of waist circumference (n=2,257)

|  |  |  | Model 4  OR (95%CI) |
| --- | --- | --- | --- |
| Total physical activity  (h/day) | <15 mg/24h  15-<30 mg/24h  ≥30 mg/24h | Q1  Q2  Q3  Q4  Q1  Q2  Q3  Q4 | Reference  Reference  **0.59 (0.40; 0.87)**  **0.67 (0.45; 0.99)**  **0.64 (0.42; 0.99)**  Reference  0.81 (0.53; 1.26)  0.66 (0.41; 1.08)  1.01 (0.62; 1.66) |
| Lower intensity physical activity  (h/day) | <15 mg/24h  15-<30 mg/24h  ≥30 mg/24h | Q1  Q2  Q3  Q4  Q1  Q2  Q3  Q4 | Reference  Reference  1.01 (0.69; 1.49)  0.80 (0.54; 1.20)  0.84 (0.55; 1.27)  Reference  1.18 (0.75; 1.87)  **0.60 (0.36; 0.99)**  1.23 (0.75; 2.01) |
| Higher intensity physical activity  (10 min/day) | <15 mg/24h  15-<30 mg/24h  ≥30 mg/24h |  | Reference  1.00 (0.91; 1.10)  0.95 (0.85; 1.07) |
| Sedentary time  (h/day) | <15 mg/24h  15-<30 mg/24h  ≥30 mg/24h |  | Reference  **1.11 (1.00; 1.22)**  1.07 (0.96; 1.20) |
| Sedentary breaks  (10/day) | <15 mg/24h  15-<30 mg/24h  ≥30 mg/24h |  | Reference  0.97 (0.80; 1.16)  0.91 (0.74; 1.12) |
| Prolonged sedentary bouts  (#/day) | <15 mg/24h  15-<30 mg/24h  ≥30 mg/24h |  | Reference  1.02 (0.88; 1.18)  1.03 (0.87; 1.21) |
| Average sedentary bout duration  (min) | <15 mg/24h  15-<30 mg/24h  ≥30 mg/24h |  | Reference  1.02 (0.98; 1.07)  1.02 (0.97; 1.07) |

*Note:* The odds ratios (OR) represent the odds of having a urinary albumin excretion of 15-<30 mg/24h or a urinary albumin excretion of ≥30 mg/24h (with a urinary albumin excretion of <15 mg/24h as reference category), respectively, relative to the odds in the first quartile for total physical activity and LPA, or per one unit increase in HPA or the sedentary behavior variables. Boldface indicates statistical significance (P <0.05). The associations in models 4 were adjusted for age, sex, glucose metabolism status, waking time, educational level, smoking behavior, alcohol consumption, energy intake, comorbid disease, mobility limitation, HPA (for the sedentary behavior variables only), sedentary time (for HPA and the sedentary behavior pattern variables only), office systolic blood pressure, use of antihypertensive medication, body mass index, total-to-HDL cholesterol ratio, triglycerides, use of lipid-modifying medication, prevalent cardiovascular disease. All analyses were based on complete cases (n=2,257). Distribution of participants according to albuminuria categories: <15 mg/24h n=1604, 15-<30 mg/24h n=216, ≥30 mg/24h n=176.

Abbreviations: CI, confidence interval; eGFR_crcys_, estimated glomerular filtration rate based on serum creatinine and serum cystatin C; HPA, higher intensity physical activity; HDL cholesterol, high-density lipoprotein cholesterol, N/A, not applicable.
